# Anxiety and Risk of Vascular Dementia in an Elderly Community Sample: The Role of Sex

**DOI:** 10.3390/brainsci10050265

**Published:** 2020-04-30

**Authors:** Javier Santabárbara, Beatriz Villagrasa, Raúl Lopez-Anton, Concepción De la Cámara, Patricia Gracia-García, Antonio Lobo

**Affiliations:** 1 Department of Preventive Medicine and Public Health, Universidad de Zaragoza, 50009 Zaragoza, Spain; jsantabarbara@unizar.es; 2 Instituto de Investigación Sanitaria de Aragón (IIS Aragón), 50009 Zaragoza, Spain; rlanton@unizar.es (R.L.-A.); conchidlc@hotmail.com (C.D.l.C.); alobosat@gmail.com (A.L.); 3 Centro de Investigación Biomédica en Red de Salud Mental (CIBERSAM), Ministry of Science and Innovation, 28029 Madrid, Spain; pgracia@salud.aragon.es; 4 Psychogeriatry Area, CASM Benito Menni, Sant Boi del Llobregat, 08830 Barcelona, Spain; 5 Department of Psychology and Sociology, Universidad de Zaragoza, 50009 Zaragoza, Spain; 6 Psychiatry Service, Hospital Clínico Universitario Lozano Blesa, 50009 Zaragoza, Spain; 7 Department of Medicine and Psychiatry, Universidad de Zaragoza, 50009 Zaragoza, Spain; 8 Psychiatry Service, Hospital Universitario Miguel Servet, 50009 Zaragoza, Spain

**Keywords:** anxiety, vascular dementia, risk factor, ZARADEMP, community study

## Abstract

*Background:* To assess the association between anxiety and risk of vascular dementia (VaD), as well as potential sex differences, in a community-based cohort. *Methods:* A random sample of 4057 dementia-free community participants aged 55 or older, from the longitudinal, community-based Zaragoza Dementia and Depression Project (ZARADEMP) study were followed for 4.5 years. Geriatric Mental State B (GMS)-Automated Geriatric Examination for Computer Assisted Taxonomy (AGECAT) was used for the assessment and diagnosis of anxiety, and a panel of research psychiatrists diagnosed the incident cases of VaD according to DSM-IV (*Diagnostic and Statistical Manual of mental disordes*). Multivariate survival analysis with competing risk regression model was performed. *Results:* In men, the incidence rate of VaD was significantly higher among anxiety subjects compared with non-anxiety subjects (incidence rate ratio (IRR) (95% confidence interval (CI)): 3.24 (1.13–9.35); *p* = 0.029), and no difference was observed in women (IRR (95%CI): 0.68 (0.19–2.23); *p* = 0.168). In the multivariate model, for men, cases of anxiety had 2.6-fold higher risk of VaD (subdistribution hazard ratio (SHR): 2.61; 95%CI: 0.88–7.74) when all potential confounding factors were controlled, with no statistical significance (*p* = 0.084), but a clinically relevant effect (Cohen’s d: 0.74). No association was found in women. *Conclusions:* In men, but not in women, risk of VaD was higher among individuals with anxiety, with a clinically relevant effect. Potential anxiety-related preventive interventions for VaD might be tailored to men and women separately.

## 1. Introduction

The analysis of risk factors of overall dementia and its most frequent types, Alzheimer disease (AD) and vascular dementia (VaD), has been put at stake in a report that includes psychological factors such as depression and anxiety [1]. In relation to this, our research group has recently reported that clinically relevant anxiety was associated with an almost four-fold increase in the risk of AD [2]. VaD is the second most frequent type of dementia, corresponding to about 20% of cases [1], with the incidence rates increasing with age, although with a less marked exponential increase than AD [3]. Most studies have suggested that men are at greater risk of developing VaD, and the risk profile has been suggested to differ in men and women [4,5].

Although it has been shown that depression is a risk factor for VaD [6], the effect of anxiety on this risk is still under study. In fact, a recent meta-analysis found only two epidemiological studies [7,8] reporting that anxiety may be associated with an increased risk for VaD [9]. Both studies were carried out in a middle-age population, and only one of them was prospective [7]. Therefore, whether anxiety in late-life is an independent risk factor for VaD remains unknown. As establishing this has implications for the development of strategies targeting dementia and is best done with cohort studies, especially in older people [10,11], new community-based longitudinal studies are needed to elucidate whether anxiety is a risk factor for VaD.

In this context, the aim of the present study was to assess the association between anxiety and risk of VaD in a community-dwelling cohort. In view of the different profile of risk factors of VaD between women and men previously reported [4,5], the analysis was stratified by sex.

## 2. Materials and Methods

This work follows the STROBE [12] and SAMPL [13] guidelines for reporting observational studies in epidemiology and statistics, respectively.

### 2.1. Sample and Procedure

We used data from the Zaragoza Dementia and Depression Project (ZARADEMP), a longitudinal, population-based study intended to document the incidence and risk factors of somatic and psychiatric diseases in adults aged ≥55 years. The Ethics Committee of the Institutional Review Board in our institutions (CEICA) approved the study according to Spanish Law, and the principles of written informed consent, privacy, and confidentiality have been maintained throughout the project.

The methods have been described in detail previously [14,15]. The representative sample was drawn from Spanish official census lists, stratified through proportional allocation by age and sex, and included institutionalized individuals. At the baseline cross-sectional study (starting in 1994), 4803 individuals were interviewed, with the refusal rate being 20.5%. This paper reports results from the baseline study (Wave I) and two follow-up waves (Waves II and III, two and four years later, respectively). A two-phase screening procedure was implemented in each of the waves. Validated Spanish versions of international instruments were used for the assessment and included the Mini-Mental Status Examination (MMSE), the Geriatric Mental State B (GMS-B), the Automated Geriatric Examination for Computer Assisted Taxonomy (AGECAT), the History and Aetiology Schedule (HAS) (medical and psychiatric history data), Katz’s Index for basic activities of daily living (bADL’s), the Lawton and Brody scale for instrumental activities (IADL’s), and the European Studies of Dementia (EURODEM) Risk Factors Questionnaire (for medical conditions). The first phase was carried out by well-trained lay interviewers at the participants’ home, or residence if they were institutionalized. In the second phase, a psychiatrist interviewed all subjects who were considered as a probable psychiatric case or if information from the first phase was inconclusive. The instruments used and interview place were the same as in the first phase.

### 2.2. Vascular Dementia Assessment and Diagnosis

At the end of the baseline assessment (Wave I), identified cases of dementia and subcases of dementia (GMS-criteria) were excluded from the follow-up waves (Waves II and III). Validity coefficients of the Spanish version of GMS-B for dementia diagnosis were as follows: sensitivity 93.2%, specificity 89.4%, positive predictive value 66.3%, negative predictive value 98.3%, and overall misclassification 9.8%, respectively [16]. Incident dementia (including subtypes) was initially diagnosed at follow-up by a psychiatrist using the above-mentioned instruments as well as Hachinski´s scale to facilitate the distinction between VaD and Alzheimer´s disease. Final VaD diagnosis (according to DSM-IV criteria) was made by consensus, which required agreement of at least three psychiatrists on a four- member panel. The validity of this diagnostic process has also been previously shown [16]. To document the precision of the panel, a proportion of detected cases was invited to a hospital diagnostic work-up, in which neuroimaging studies and a neuropsychological battery were incorporated. Agreement on the diagnosis of dementia was 95.8%, and on the type of dementia, it was 87.5%.

### 2.3. Anxiety Assessment and Diagnosis

The diagnosis of anxiety at baseline was based on the GMS-AGECAT system. After symptom assessment, a computer program compared syndrome clusters (e.g., dementia, depression, anxiety) to reach a final diagnosis. If the AGECAT score was ≥3, the subject was considered a “case” of anxiety (clinically relevant anxiety that requires clinical intervention). If this score was 1 or 2, the subject was considered as a “subcase” of anxiety (subsyndromal or mild anxiety, and if this score was 0, the subject was considered a “non-case” or unaffected). Subsyndromal anxiety and anxiety symptoms have been associated with significant functional impairment [17,18,19,20], highlighting their clinical importance. Unlike other previous studies, [2,15], in view of the small sample of “cases” (*n* = 91) and the low expected incidence of vascular dementia [1], we merged the “case” (*n* = 91) and “subcase” (*n* = 1645) categories for the purposes of this paper.

### 2.4. Ascertainment of Mortality

A reliable source, the official population registry in the city of Zaragoza, was reviewed to ascertain all-cause mortality of the ZARADEMP respondents. Information in the registry was completed and verified via death certificate, which provides accurate information, including day, month, and year of death. Days from birth to the date of death were calculated for each subject, and those individuals remaining alive or missing at the end of follow-up (emigrated, not available) were included in the analysis as censured.

### 2.5. Covariates

We included in the analysis the following covariates: socio-demographic characteristics (age, sex, educational level, marital status, and living alone), medical risk factors (vascular disease (angina, myocardial infarct, and/or stroke), diabetes, and hypertension), health status, depression, and cognitive status [15]. Additionally, we specifically included vascular risk factors (smoking, statin use, body mass index, and alcohol intake) based on the medical history obtained with the EURODEM Risk Factors Questionnaire [21].

We distinguished three categories at the educational level: “illiterate” (cannot read or write, and/or <2 years of formal education), “primary” (incomplete or complete), and “secondary school or higher”. Concerning marital status, a participant could be “single”, “married or living as a couple”, “divorced or separated”, or “widowed”. For medical risk factors, vascular diseases was dichotomized, differentiating between the presence of angina, miocardyal infarct and/or stroke, and no history of vascular disease. Diabetes was categorized in subjects receiving treatment for diabetes or previous medical history of diabetes and no diabetes. Blood pressure (BP) was categorized as hypertension if BP was >140/90 or the individual was in treatment for hypertension or absence of hypertension. During the interview, BP was measured using a standard manual tensionmeter, selecting the average of two measurements. Health status was classificated following HAS-criteria, and we distinguished “good health” (physical illness absent) or “not good” (if a physical illness was present). Depression was registered according to the AGECAT diagnosis.

### 2.6. Statistical Analysis

The analysis was performed stratified by sex. Incidence rate and incidence rate ratio (IRR) were calculated with standard procedures. In a first step of the survival analysis, we built the cumulative incidence functions (CIF) for the anxiety disorder groups to estimate the probability of incident VaD, taking into account the competing event (death) as time progressed. Then, we used the Fine and Gray multivariate (competing risk) regression model to calculate participants’ risk of experiencing VaD, taking into account the competing event (death) as time progressed, with age as timescale with delayed entry.

Cohen’s d was calculated to document differences in risk of VaD between anxiety groups. This coefficient measures the effect size, and may be especially relevant in cases of small samples, when the differences found do not reach statistical significance. The effect size for the hazard ratio (HR) was classified as small (~0.2), medium (~0.5), or large (~0.8) [22].

Statistical analyses were conducted using *R* software (https://www.r-project.org/).

## 3. Results

**Figure 1:**
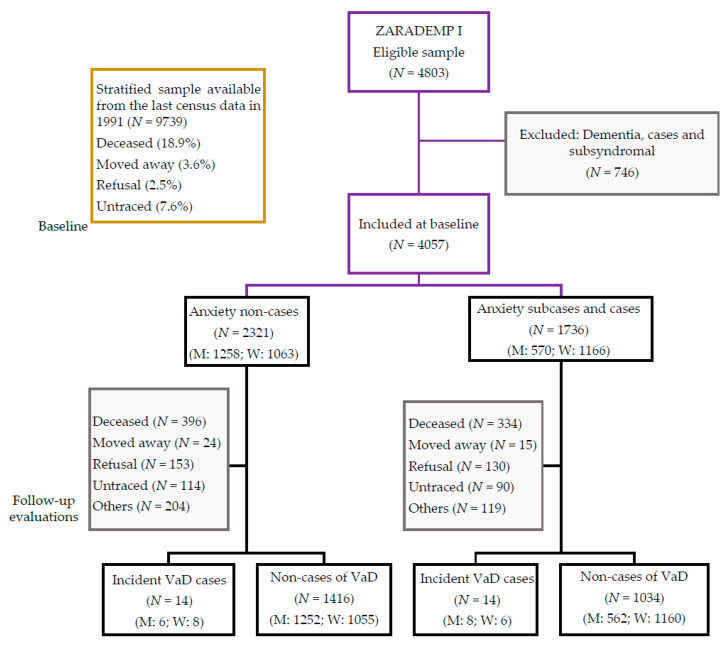
Zaragoza Dementia and Depression Project (ZARADEMP) study flow chart. *Notes*: M: men; W: women. VaD: vascular dementia.

Figure 1 illustrates the flow diagram of ZARADEMP. Our final sample for the 4.5 year follow-up (median 4.4 years; interquartile range: 3.0–4.9 years) included 4057 participants without any type of dementia or cognitive impairment at baseline.

Baseline characteristics according to anxiety status have been described elsewhere [15]. In brief, compared with non-cases, cases and subcases of anxiety were more likely to be female, to report poor health status, and to have depression and/or disabilities for instrumental ADLs.

Table 1 shows baseline demographic and clinical characteristics according to VaD incidence status. The incident VaD group was significantly older, more likely to perform worse cognitively, and more likely to have hypertension and vascular disease. Statistically significant differences were observed for age and MMSE scores both in men and women, and for vascular disease in men. Participants lost during follow-up were significantly older and more likely to be illiterate than those re-evaluated; the MMSE scores were also lower among those lost, as we reported previously [15].

As seen in Table 2, 14 incident male cases of vascular dementia were found in the follow-up assessment waves (8 cases of anxiety (1.1%) and 6 non-cases of anxiety (0.5%)). The incidence rate was significantly higher among cases of anxiety (2.1 per 1000 person-years) relative to non-cases of anxiety (0.6 per 1000 person-years) (incidence rate ratio, IRR (95% confidence interval (CI)): 3.24 (1.13–9.35); *p* = 0.029) (Table 2). No difference in incidence rate of vascular dementia according to anxiety status at baseline was observed in women (Table 2).

**Figure 2:**
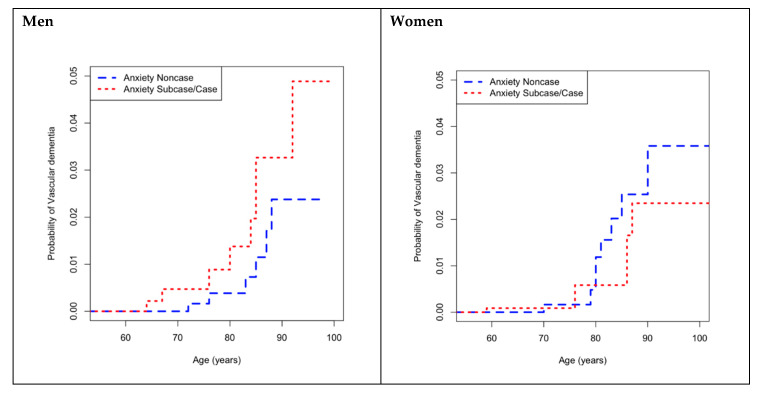
Cumulative incidence functions for incident VaD according to anxietystraitified by sex. VaD: vascular dementia.

Crude comparison of the CIF by anxiety status in men showed that, at the 4.5 year follow-up, compared with the non-cases of anxiety, the probability of developing vascular dementia was higher for all ages, in the cases of anxiety (Figure 2). For example, for men aged 85 years, the probability (in percentage) of VaD in the cases group was 2.0% (95% CI (0.7–4.5)), higher than in the non-cases group (0.7%; 95% CI (0.2–2.1)). No difference in probability of developing vascular dementia according to anxiety status at baseline was observed in women (Figure 2).

Table 2 also shows the results of the competing risks regression analysis of incident vascular dementia associated with anxiety status, stratified by sex. For men, cases of anxiety had a 2.6-fold higher risk of vascular dementia when all potential confounding factors were controlled (subdistribution hazard ratio (SHR) = 2.61; 95% CI (0.88–7.74)). This effect tended to be significant (*p* = 0.084) and was close to the medium magnitude (Cohen’s d = 0.74). No association between cases of anxiety at baseline and vascular dementia incidence was found in women.

## 4. Discussion

Concerning the objectives, our study has found that the incidence rate of VaD in men, but not in women, was significantly higher among cases of anxiety relative to non-cases of anxiety, with the IRR being more than three-fold higher. Moreover, and specifically, the risk of VaD was 2.6-fold higher in men’s anxiety cases compared with the non-case category when controlling for sociodemographic characteristics, vascular risk factors, health status, depression, and cognitive status at baseline. This association was not significant, but the effect size was almost medium, that is, this effect is clinically significant [22]. By contrast, the increased risk of VaD was not found for women’s anxiety cases. To our knowledge, this is the first study addressing independently in men and women the association between anxiety and VaD risk in a community-dwelling cohort of older people.

Whereas depression has been broadly studied as a risk factor for VaD, showing a positive association [6], previous evidence in the study of anxiety as a risk factor of VaD is scarce, and the results are difficult to compare with ours because of methodological differences. A recent meta-analysis concluded, in consonance with our study, that anxiety increases by 88% the risk of VaD, although the magnitude of the effect found was higher in our sample [9]. However, only two studies could be included in this meta-analysis and, contrary to ours, both assessed anxiety starting in mid-life. One of them, Zilkens et al.’s [8] report, was a population-based case-control study using state-wide hospital inpatient, outpatient mental health, and emergency records of patients diagnosed with ICD (*International Classification of Diseases*) criteria [8]. The other study, by Gallacher et al. [7], a longitudinal 17 year follow-up, was limited to men, and anxiety was assessed with the STAI (*State- Trait Anxiety Inventory*) scale [7]. Therrien and Hunsley [23] have concluded that these types of instruments to assess anxiety lack sufficient evidence to warrant their use with older adults, contrary to the GMS-AGECAT [24,25], used in our study, which was developed for investigations in the older community.

To our knowledge, our study is the first one using a competing risk model [26]. This is an advantage over traditional models (e.g., Kaplan–Meier and Cox regression), as they do not take into account competing risks of death, and may thus overestimate the risk of disease in the presence of high rates of mortality. Consequently, this method is particularly relevant in studies of older people [27]. In support of this, a secondary analysis in this study has shown that, according to the Kochar–Lam–Yip test [28], the risk of death was significantly higher than the risk of VaD (*p* < 0.001, data not shown). Moreover, when controlling for the effect of age in the risk of VaD at baseline, and in contrast with previous studies using a time-on study and including age as a covariate in the regression models [7,8], we used exact age as the time-scale. The exact age is preferred as a time-scale to avoid bias in effect estimates in samples of older adults because age is strongly associated with some covariates (e.g., chronic diseases) [29]. We have previously documented, in this same sample, that dementia risk increases significantly with an individual’s age [14].

Vascular dementia may be the result of a single strategic infarct, multiple cortical or lacunar infarcts, or microvascular insults to regions of the brain critical for cognitive function [30]. Most studies have suggested that both the prevalence and incidence of VaD [4,31,32] as well as the frequency of vascular risk factors for VaD (atrial fibrillation, heart failure, high blood pressure, atherosclerosis, obesity, and diabetes), are higher in men when than in women [4]. Therefore, it might be argued that this increased vulnerability to VaD among men might have significantly influenced the main findings in this study, namely, the increased incidence of VaD and the increased risk of VaD among anxiety cases in men. However, as we carefully controlled for vascular risk factors, our results suggest that anxiety could be an independent risk factor.

The association of anxiety and increased risk of VaD in men could be explained by various mechanisms. Firstly, anxiety is associated with an elevated risk of a range of different cardiovascular events, including stroke, coronary heart disease, heart failure, and cardiovascular death [33]. These cardiovascular events are, in turn, risk factors for VaD [30] and, as we have noted, are more frequent in men [4]. Secondly, anxiety might promote negative neuroplasticity, as suggested by Vance et al. [34], thereby decreasing the cognitive reserve. Thirdly, it has been suggested that anxiety may induce accelerated aging across multiple biological processes [35]. Recent research of cellular ageing indicates that the human plasma peptide Gly-His-Lys, which has powerful anti-anxiety effects, is related to the suppression of several molecules that favor accelerated cellular ageing [36,37]. Fourthly, anxiety is linked to elevated levels of glucocorticoids [38], which have been shown to increase cardiovascular disease risk [39], and to be associated with impaired cognitive performance [40], as well as with impaired memory retrieval and impaired working memory during emotionally arousing test situations [41]. Lastly, recent studies on gut-microbiota have shown that anxiety is associated with increased gut permeability [42] and with changes in gut-microbiota composition [43]. Gut microbiota has been suggested to play a role in the modulation of cognitive function, and the possibility that it is linked to frailty and dementia in older people has been hypothesized [44].

One question that could be posed is whether anxiety is a true risk factor or a prodromal syndrome of VaD. Controversy has surrounded this issue in relation to overall dementia [45], but we have previously supported the view that it is an independent risk factor [15], coinciding with the conclusions of a recent systematic review [46]. Although we are cautious in interpreting the results in this study given the limited number of cases of anxiety, we believe our results similarly support the notion of increased risk. First, we excluded at baseline all those individuals with mild cognitive deficits (“subsyndromal” dementia) to minimize the possibility of including in the cohort individuals with prodromal cognitive deficits; and second, cognitive status at baseline was controlled in the statistical analysis.

Our study had other strengths, such as the inclusion of a representative population sample that also contains institutionalized individuals, the use of international instruments revalidated in our specific population, and the fact that the AGECAT diagnostic system has been shown to have clinical relevance in older people [47]. We also included actual mortality data in Fine and Gray’s model to study anxiety as a risk factor for VaD. We believe the consideration of risk and protective factors in men and women separately may accelerate etiological research in the field of dementia and VaD. The results of the study may imply that future preventive interventions for VaD related to anxiety could be tailored to men and women separately [48].

Some limitations must also be noted. Hospital-based diagnosis was not completed in all cases of dementia, and we did not have any data on apolipoprotein E. In contrast to other previous studies [2,15], in this case, we did not differentiate between cases and subcases of anxiety owing to the low incident cases of vascular dementia (n = 14), which prevents comparison between anxiety subgroups. Thus, statistical power was low, and statistical significance was not reached. Furthermore, we did not control for the use of psychotropic medication, and some studies have associated the use of psychotropics with a higher risk of dementia [49]. We did not include statin use in the models, but none of the incident cases of VaD (men or women) took them. Depression was not included in the male models because none with incident VaD suffered depression at baseline; similarly, tobacco use was not included in the female models because no women with incident VaD smoked.

## 5. Conclusions

In conclusion, even after controlling for critical risk factors in older individuals, including vascular risk factors, depression, and cognitive status, the incidence rate of VaD in men, but not in women, was significantly higher (more than three-fold higher) among anxiety individuals relative to non-anxiety individuals. Specifically, the risk of VaD was 2.6-fold higher in ‘anxiety’ men compared with those in the ‘non-case’ category. Whereas this association did not reach statistical significance, the effect size was medium, that is, this effect is clinically significant. This association was not found for women. The results of the study may have implications for the prevention of VaD.

## Figures and Tables

**Table 1:** Baseline characteristics according to incident VaD status stratified by sex.

		Men (*n* = 1828)		Women (*n* = 2229)		
	No VaD (*N* = 1814)	Incident VaD (*N* = 14)	*p*-Value	No VaD (*N* = 2215)	Incident VaD (*N* = 14)	*p*-Value
Age (years)	71.7 (SD = 9.1)	80.3 (SD = 8.2)	<0.001	72.3 (SD=9.1)	79.8 (SD = 7.9)	0.002
Education			0.718			
Primary school	1282 (71.4%)	11 (78.6%)		1714 (77.9%)	12 (85.7%)	0.142
High school or higher	411 (22.9%)	2 (14.3%)		277 (12.6%)	0 (0%)	
Marital status			0.100			0.493
Married/with partner	1426 (78.9%)	9 (64.3%)		1088 (49.2%)	5 (35.7%)	
Divorced/separated/widowed	258 (14.3%)	5 (35.7%)		871 (39.8%)	8 (57.1%)	
Living alone	165 (9.1%)	1 (7.1%)	0.800	532 (24.0%)	3 (21.4%)	0.821
Body mass index (kg/m^2^)	26.5 (SD = 4.9)	27.9 (SD = 3.8)	0.279	26.9 (SD = 6.8)	27.7 (5.2)	0.654
Current smoking	486 (26.8%)	1 (7.1%)	0.131	71 (3.2%)	0 (0%)	1
Current alcohol consumption	730 (40.3%)	6 (42.9%)	0.848	194 (8.8%)	1 (7.1%)	0.830
Hypertension	1170 (64.7%)	12 (85.7%)	0.158	1553 (70.1%)	12 (85.7%)	0.254
Diabetes	224 (12.5%)	1 (7.1%)	0.845	274 (12.5%)	2 (14.3%)	0.691
Statin use	93 (5.1%)	0 (0%)	1	145 (6.5%)	0 (0%)	1
Vascular disease	250 (13.8%)	6 (46.2%)	0.005	201 (9.1%)	2 (14.3%)	0.369
Health status (‘not good’)	906 (49.9%)	9 (64.3%)	0.423	1177 (53.3%)	9 (64.3%)	0.437
Depression	93 (5.1%)	0 (0%)	1	359 (16.2%)	3 (21.4%)	0.486
MMSE score	27.5 (SD = 2.6)	26.1 (SD = 2.0)	0.043	27.0 (SD = 2.5)	25.4 (SD = 2.6)	0.018

*Notes*: VaD: vascular dementia. Data are given as means (standard deviation) or number (%). MMSE = Mini-Mental State Examination.

**Table 2:** Anxiety status and risk of VaD stratified by sex.

				Univariate Model		Multivariate Model	
Anxiety Status at Baseline	No. (%) of VaD Incident Cases	Person-Years	IRR (95% CI)	SHR (95% CI)	*p*-Value	SHR (95% CI)	*p*-Value
*Men*							
Non-case (*n* = 1258)	6 (0.47)	4938	1	1		1	
Subcase/Case (*n* = 570)	8 (1.40)	2107	3.24 (1.13–9.35)	2.37 (0.81–6.88)	0.110	2.61 (0.88–7.74)	0.084
*Women*							
Non-case (*n* = 1063)	8 (0.75)	4261	1	1		1	
Subcase/Case (*n* = 1166)	6 (0.51)	4706	0.68 (0.19–0.23)	0.72 (0.25–2.09)	0.550	0.70 (0.25–1.99)	0.500

Notes: VaD: vascular dementia. IRR: incidence rate ratio. SHR: subdistribution hazard ratio. aReported SHR of VaD is related to non-cases, confidence intervals (CIs) and p-values related to SHR were from “normal approximation” of Wald’s χ2 test with 1 df. The univariate model included anxiety status. The multivariate model included terms for sociodemographic characteristics (education, marital status, and living alone), vascular risk factors (body mass index, smoking (only in men), alcohol consumption, previous vascular disease, hypertension, and diabetes), health status, depression (only in women), and cognitive status at baseline (MMSE).
